# Esophagus Impaction and Perforation by Jujube: A Tale of Double Trouble

**DOI:** 10.34172/mejdd.2025.446

**Published:** 2025-10-31

**Authors:** Ahmad Shukri Mohamad, Ikhwan Sani Mohamad, Michael Wong Pak Kai, Wan Mokhzani Wan Mokhter, Maya Mazuwin Yahya, Siti Rahmah Hashim Isa Merican

**Affiliations:** ^1^Department of Surgery, Universiti Sains Malaysia, Kubang Kerian, Kelantan, Malaysia; ^2^Hospital Pakar Universiti Sains Malaysia, Kubang Kerian, Kelantan, Malaysia

**Keywords:** Jujube, Oesophageal impaction, Oesophageal perforation, Flexible endoscopy, Rigid endoscopy

## Abstract

Sharp foreign body ingestion may result in simple impaction to mucosal perforation along the gastrointestinal tract. We report a rare case of a 62-year-old woman with jujube ingestion, a fruit that contains sharp-edged seeds causing impaction in the esophagus. Contrast computed tomography (CT) confirmed the transverse lie of the seed at the esophageal wall with features of through-and-through perforation. Flexible endoscopy under local anaesthesia successfully retrieved the jujube seed, followed by application of endoscopic hemoclips for the esophageal perforation. The patient was discharged well within a few days with no fatal outcomes.

## Introduction

 Sharp foreign body ingestion may cause simple impaction, intraluminal to mucosal perforation, and is considered a surgical emergency. Jujube fruit is classified as a sharp foreign body. It contains a spindle-shaped seed with pointy, sharp ends, which may easily get impacted anywhere along the lumen of the gastrointestinal tract.^1^ Jujube seed impaction is common; however, the incidence of organ perforation is rare.^1^ The fruit is always consumed in eastern countries, especially China, as it has many benefits for health. According to Mei and colleagues,^1^ the incidence is high, specifically in the northern part of China. In South East Asia, particularly Malaysia, the occurrence of complications due to jujube ingestion has yet to be reported.

 Hence, we report our first-hand thrilling experience in managing jujube seed impaction and perforation of the esophagus in our centre. To this age, there are no consensual guidelines for the management of esophageal perforation due to sharp foreign body impaction. European Society of Gastrointestinal Endoscopy (ESGE) recommended plain radiography for unknown foreign body ingestion, and computed tomography (CT) scan for all patients suspicious of perforation.^2^ The treatment, however, varies from choosing an endoscopic approach between rigid endoscopy (RE) and flexible endoscopy (FE) or surgery, depending on the nature of the foreign body, location of impaction, and complications. ESGE recommended urgent (within 2 hours to 6 hours) therapeutic esophagogastroduodenoscopy for sharp-pointed objects or batteries in the esophagus.^2^ For our case, the FE approach became the ideal choice to perform two tasks: retrieve the foreign body and primary closure of the perforation site.

## Case Report

**Figure 1:**
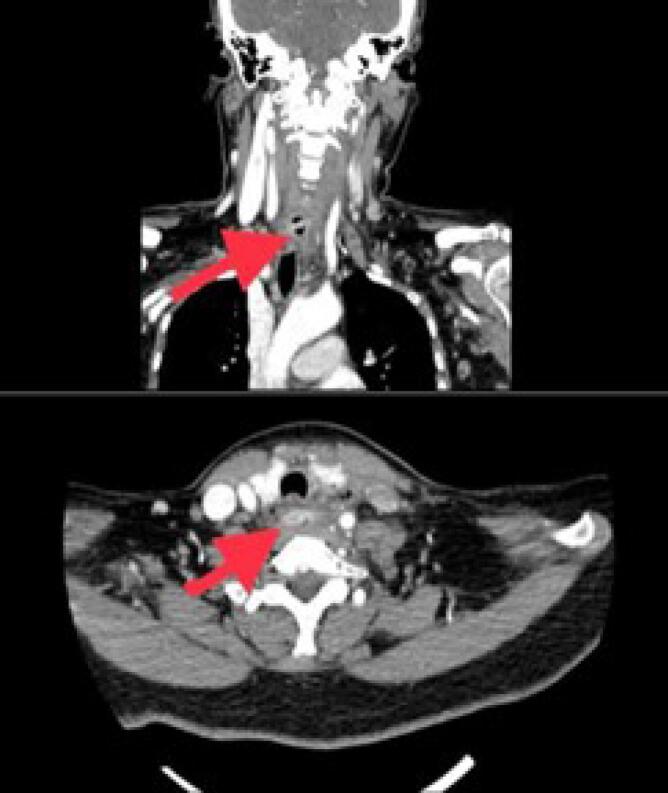
Sagittal and axial views of CECT neck showed an impacted jujube seed in the esophagus

 A 62-year-old woman with underlying hypertension went to the emergency department complaining of odynophagia with foreign body sensation in her neck. She had a history of jujube fruit ingestion earlier on that day. Physical examination elicited tenderness around the neck. Plain neck radiography noted air trapping at the level of C6/C7 vertebrae. However, she refused further treatment and was discharged home at her own risk. Two days later, she presented again to the emergency department with worsening odynophagia and fever. Neck examination showed tenderness at the anterior and lateral neck. Her vital signs were otherwise stable. Contrast-enhanced CT (CECT) neck confirmed an elongated foreign body in transverse lie within the esophagus with adjacent air pockets and enhancing soft tissue density at the peri-esophageal region, suggestive of perforation (Figure 1).

**Figure 2:**
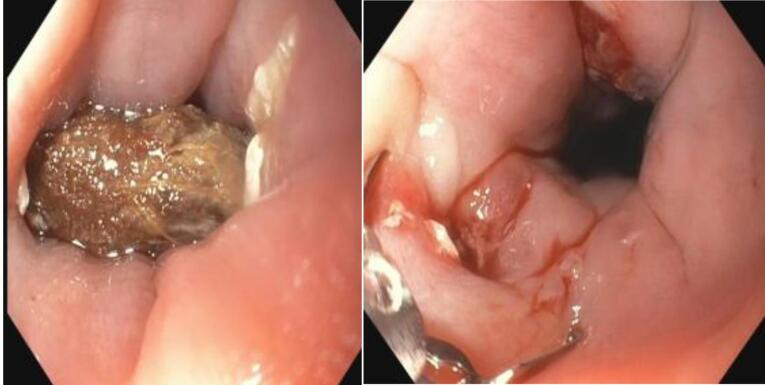
(A) Impaction in the esophagus with transverse lie. (B) Through-and-through perforation at the esophageal wall after seed removal

**Figure 3:**
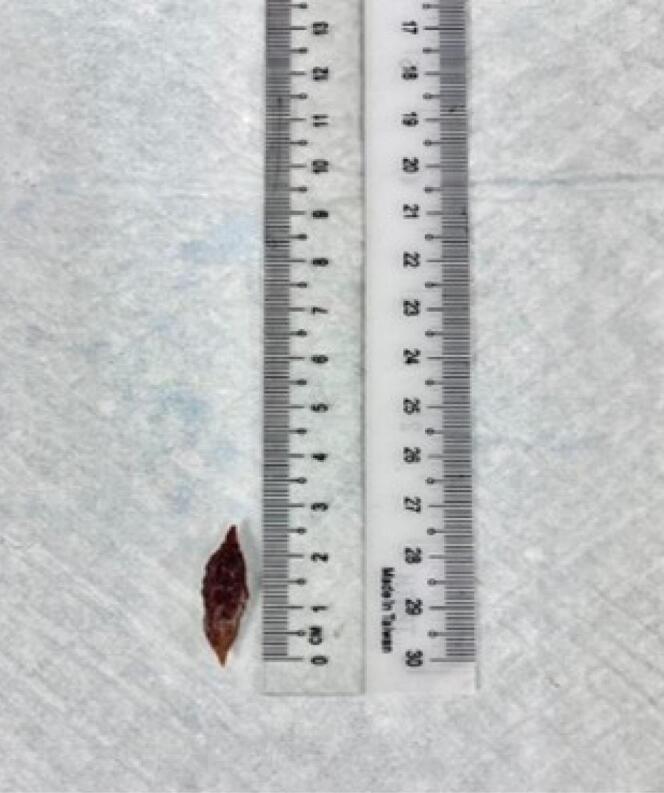
Jujube seed with sharp edges

**Figure 4:**
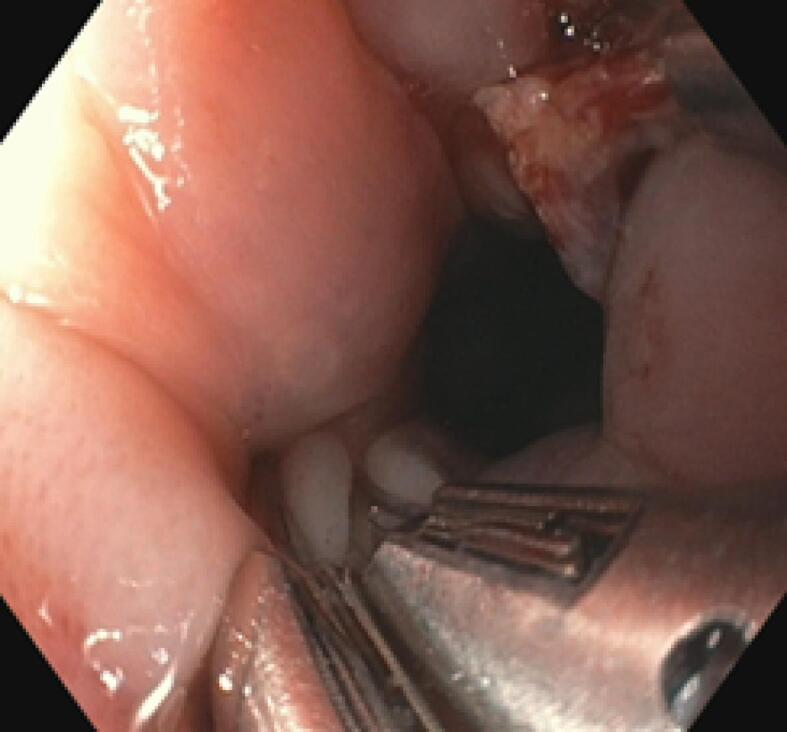
Perforation area closed by hemoclips

 Prompt intervention was done with flexible endoscopy under local anaesthesia. Endoscopically, the seed was found stuck in a transverse lie within the oesophageal lumen 15cm from the incisor (Figure 2A). Manipulation was done using rat tooth forceps until both edges dislodged from the esophageal mucosa. The seed was successfully retrieved using an endoscopic basket (Figure 3). Inspection of the impacted site revealed a through-and-through mucosal perforation with slough and surrounding edema (Figure 2B). Both perforation areas were clipped three times using endoscopic hemoclips (Figure 4). The procedure was completed in 44 minutes. The patient was stable throughout the procedure with no life-threatening complications.

## Discussion

 ESGE has classified swallowed foreign bodies into five subtypes: blunt objects, sharp-pointed objects, long objects, food boluses, and others.^2^ Jujube is classified as a sharp-pointed object, as it is a rounded red color fruit with a sharp-pointed seed inside.^3^ Due to these features, it is easily impacted along any gastrointestinal tract lumen when accidentally ingested.

 A retrospective study concluded that jujube seeds most commonly lodge in the esophagus.^4^ Prolonged impaction may worsen the severity of the event. Another study showed that impaction for more than 24 hours had a damaging effect on the esophageal mucosa, leading to perforation.^5^ The motive of swallowing the seed is not known; however, there was a study that reported the incident was mostly accidental.^6^

 Managing oesophageal perforation caused by jujube seed needs a meticulous strategy with well-executed intervention to remove the foreign body and treat the perforation site without further causing injury to the surrounding area. Due to its pointy edges, any attempt to remove the seed might worsen the perforation site if no proper precautions. To date, no standard protocol has been outlined to facilitate the treatment. For removal of foreign body, ESGE recommended an endoscopic approach as the first choice of intervention, preferably within 2 hours, but at the latest within 6 hours ^2^

 Endoscopic attempt can be either FE or RE.^7^ The choice between FE versus RE, however, depends on several factors such as age and clinical condition of the patient, location of the ingested foreign body, and primarily surgeon preference. A study outlined that FE and RE showed similar outcomes, only FE is preferable due to shorter procedural time, better visual, and can be performed under local sedation.^7,8^ Meanwhile, some authors mentioned RE is better as it has a good grasp of the jujube seed because of the larger forceps.^1^

 Apart from that, flexible endoscopy usage has been increasing in trend as it is cost-effective, against general anaesthesia, and practical.^8-10^ Several retrieval forceps are available for FE dealing with sharp foreign bodies: rat-tooth, alligator-tooth, or shark-tooth.^10^ ESGE again stated FE is the best therapeutic choice for foreign body impaction in the upper gastrointestinal tract.^2^ Although FE is favorable than RE, meta-analysis by Ferrari D and coworkers concluded both FE and RE yielded similar success rate and safety.^8^

 Regarding oesophageal perforation, surgery remains the mainstay of therapy. However, endoscopic repair via multiple techniques, such as clipping and stenting, has expanded as a modality of treatment pertaining to esophageal perforation.^11^ Endoscopic clipping was first demonstrated in 1995; subsequently, the trend has been higher in recent years. A study highlighted that endoscopic clips are safe and suitable for small perforations with an average of 1cm.^12^ For our case, we preferred FE as a first-line approach and successfully retrieved the jujube seed without causing further structural damage to the esophageal mucosa under local anaesthesia. Application of rat tooth forcep with delicate manipulation was able to dislodge one of its pointy edges, pulling the edges out entirely, and placed on the mucosa before retrieval using a basket. The perforation site of the oesophageal wall could be visualized and clipped using three hemoclips. Adjunct management with parenteral nutrition was started on the patient for one week till re-evaluation of the perforation site was done with a barium study to establish no leakage.

## Conclusion

 Esophageal perforation caused by jujube seed impaction is fatal if no prompt intervention is taken. Endoscopic approach, especially flexible endoscopy, has proven beneficial as a first-line retrieval procedure and is recommended as a therapeutic strategy.
